# Isobavachalcone exhibits antifungal and antibiofilm effects against *C. albicans* by disrupting cell wall/membrane integrity and inducing apoptosis and autophagy

**DOI:** 10.3389/fcimb.2024.1336773

**Published:** 2024-01-22

**Authors:** Weidong Qian, Jiaxing Lu, Chang Gao, Qiming Liu, Wendi Yao, Ting Wang, Xiaobin Wang, Zhifeng Wang

**Affiliations:** ^1^School of Biological and Pharmaceutical Engineering, Shaanxi University of Science and Technology, Xi’an, China; ^2^Department of Urology, Henan Provincial People’s Hospital, Zhengzhou University People’s Hospital, Zhengzhou, China; ^3^Department of Urology, Southern University of Science and Technology Hospital, Shenzhen, China

**Keywords:** Isobavachalcone, anti-candida, antibiofilm, apoptosis, autophagy

## Abstract

Isobavachalcone (IBC) is a natural flavonoid with multiple pharmacological properties. This study aimed to evaluate the efficacy of IBC against planktonic growth and biofilms of *Candida albicans* (*C. albicans*) and the mechanisms underlying its antifungal action. The cell membrane integrity, cell metabolic viability, and cell morphology of *C. albicans* treated with IBC were evaluated using CLSM and FESEM analyses. Crystal violet staining, CLSM, and FESEM were used to assess the inhibition of biofilm formation, as well as dispersal and killing effects of IBC on mature biofilms. RNA-seq combined with apoptosis and autophagy assays was used to examine the mechanisms underlying the antifungal action of IBC. IBC exhibited excellent antifungal activity with 8 μg/mL of MIC for *C. albicans*. IBC disrupted the cell membrane integrity, and inhibited biofilm formation. IBC dispersed mature biofilms and damaged biofilm cells of *C. albicans* at 32 μg/mL. Moreover, IBC induced apoptosis and autophagy-associated cell death of *C. albicans*. The RNA-seq analysis revealed upregulation or downregulation of key genes involved in cell wall synthesis (*Wsc1* and *Fks1*), ergosterol biosynthesis (*Erg3*, and *Erg11*), apoptisis (*Hsp90* and *Aif1*), as well as autophagy pathways (*Atg8*, *Atg13*, and *Atg17*), and so forth, in response to IBC, as evidenced by the experiment-based phenotypic analysis. These results suggest that IBC inhibits *C. albicans* growth by disrupting the cell wall/membrane, caused by the altered expression of genes associated with β-1,3-glucan and ergosterol biosynthesis. IBC induces apoptosis and autophagy-associated cell death by upregulating the expression of *Hsp90*, and altering autophagy-related genes involved in the formation of the Atg1 complex and the pre-autophagosomal structure. Together, our findings provide important insights into the potential multifunctional mechanism of action of IBC.

## 1 Introduction

Globally, invasive fungal diseases caused by pathogenic fungi are responsible for at least 300 million severe cases and 1.5 million deaths every year, particularly among at-risk and immunocompromised individuals. *Candida*, *Aspergillus*, and *Pneumocystis* infections accounted for 76.3% of the diagnosed fungal infections. *Candida* is the single most common cause of fungal infections worldwide, with approximately 154 species belonging to the genus *Candida*. Among these, *Candida albicans* (*C. albicans*), are the most frequent cause of nosocomial fungal infections and invasive candidiasis (Lionakis et al., 2023). In addition, in one multicenter study conducted in the United States, it was found that 44% of candidemia cases in critically ill COVID-19 patients were attributed to the *C. albicans* species (Nag and Kaur, 2021). As a commensal fungus in the human microbiome residing within and outside the body, *C. albicans* asymptomatically colonizes the shin, oral mucosa, gastrointestinal tract, and genital tract (Peroumal et al., 2022).

A major contributor to the virulence of *C. albicans* is its ability to form biofilms, which are compact packed communities of cells that are attached to a surface, consisting of yeast cells, pseudohyphae, and hyphae cells, and have properties that are remarkably different from their corresponding planktonic counterparts (Barbosa et al., 2020). A recognized feature of biofilms is their markedly increased resistance to chemical, physical, and immune response treatments, making them more difficult to treat in clinical settings (Ciofu et al., 2022). The recalcitrance of biofilms to conventional antifungal therapies and the immune system is a major cause of prolonged, recurrent, or intractable infections, leading to an increase in healthcare costs (Fan et al., 2022). Furthermore, the spread of fungal cells within biofilms has great clinical significance because they can spread from the deposition site to distant host tissues, thus triggering the establishment of a new biofilm that causes disseminated invasive diseases or candidaemia (Rumbaugh and Sauer, 2020).

Resistance to antifungal agents has emerged as a significant challenge worldwide due to the inappropriate use of antifungal drugs (Fisher et al., 2022). Moreover, the global overuse of antibiotics promotes the growth of *C. albican* by increasing the microbial ecology in humans, consequently exacerbating the multiplication and propagation of cells, followed by biofilm development (Browne et al., 2021). Mechanistically, there are four main classes of antifungal drugs currently used to treat fungal infections, azoles, polyenes, pyrimidine analogues, and echinocandins, which directly target the biosynthetic pathway of ergosterol, the fungal cell wall, or fungal DNA/RNA (Houšť et al., 2020). However, only echinocandins and liposomal formulations of AmB can be used to treat *C. albicans* biofilms. Hence, the need to exploit novel and more effective antibiofilm agents has increased several-fold, owing to the increased incidence of severe biofilm-associated infections. Recently, natural active compounds originating from medicinal plants have been exploited as promising antifungal and antibiofilm agents against *C. albicans*, owing to their abundant sources (Atanasov et al., 2021). Some studies have suggested that natural plant compounds affect important biofilm virulence factors, including the inhibition of biofilm formation, adhesion, promotion of biofilm cell detachment, and dispersion of established mature biofilms (Lu et al., 2019; Park et al., 2019). Similar to classical antifungal drugs, these natural compounds exert anti-*Candida* effects by generating reactive oxygen species (ROS) and targeting *Candida* cellular metabolic pathways, including ABC transporter mediated drug efflux, cell cycle progression, mitochondrial activity, and ergosterol, chitin, and cell wall biosynthesis (Shariati et al., 2022; Campos Péret et al., 2023).

Isobavachalcone (IBC), previously known as isobapsoralcone, is a plant-derived chalcone compound with multiple pharmacological activities, including anticancer, antibacterial, anti-inflammatory, antiviral, ant neuroprotective, and bone-protective activities (Xing et al., 2022). IBC is commonly extracted isolated from the seeds of *Psoralea corylifolia* Linn (*P. corylifolia* L.)., which is one of the most commonly used traditional medicines in China and India. *P. corylifolia* L. has been recommended in the treatment of leucoderma, psoriasis, osteoporosis, and gynecological bleeding (Wang et al., 2021). Mechanistically, the multifaceted mechanisms of action of IBC have been elucidated, including inducing the generation of ROS (Zhao et al., 2022); altering the AKT, ERK, and WNT pathways; promoting apoptosis-induced cell death through mitochondrial or endoplasmic reticulum pathways; and suppressing the production of several inflammatory mediators by activating the NRF2/HO-1 pathway (Xing et al., 2022). In addition, previous studies indicated that IBC was active against Gram-positive bacteria, mainly against Methicillin-susceptible *Staphylococcus aureus* and Methicillin-resistant *S. aureus*, with minimum inhibitory concentration (MIC) values of 1.56 and 3.12 µg/mL, respectively (Assis et al., 2022). However, the antifungal and antibiofilm efficacies of IBC against *C. albicans* remain largely unexplored. The present study aimed to investigate the antifungal and antibiofilm efficacy of IBC against *C. albicans* and its underlying mechanisms of action.

## 2 Materials and methods

### 2.1 Reagents

IBC (purity ≥ 98%) was purchased from Chengdu Lemeitian Pharmaceutical Technology Co., Ltd. (Chengdu, China). A stock solution of IBC dissolved in 10% dimethyl sulfoxide (DMSO) was sterilized using a filter membrane and diluted to the desired concentration. Several types of dyes including SYTO 9, FUN^®^1, Calcofluor^®^ White M2R stain (CWS), and propidium iodide (PI), were purchased from Invitrogen (Thermo Fisher Scientific, Waltham, Massachusetts, USA).

### 2.2 Determination of MIC and minimum fungicide concentration

The MIC of IBC against *C. albicans* SC5314 was determined using the Clinical and Laboratory Standards Institute (CLSI) M27-A2 standard method with minor modifications. Briefly, 100 μL of the final concentration of fungal suspension (1 × 10^4^ colony-forming units (CFUs)/mL) was prepared by diluting the fresh cultures (approximately 10^8^ CFU/mL) with RPMI-1640 medium (Gibco, Fisher Scientific, USA), transferred into each well of the 96-well microplate. Then a final concentration from 0.5 to 1024 μg/mL was added into individual well to yield a final volume of 200 μL per well, and further incubated at 30°C. Fugal cells treated with fresh RPMI-1640 without IBC were used as controls. After incubation for 22-24 h, the optical density (OD) of the cultures in each well was measured at 600 nm using a spectrophotometre. Meanwhile, the fungal cells of each well of a 96-well plate were serially diluted using 100 mM phosphate buffer saline (PBS, pH 7.0), spread onto YPD agar plate, and further cultured for 24 h at 30°C to calculate CFUs to determine MFC_90_ of IBC against *C. albicans* SC5314. The MIC is defined as the lowest effective concentration of IBC that inhibited the visible growth of the organism tested after 22-24 h of growth. Furthermore, the MFC_90_ is defined as the minimum concentration of IBC at which 90% inhibition of *C. albicans* SC5314 cell growth occurs after 24 h.

### 2.3 Time-kill curve assay

To investigate the effect of IBC on the growth kinetics of *C. albicans* SC5314, we cultured *C. albicans* SC5314 at the MIC of IBC according to a previously described method (Appiah et al., 2017). Briefly, overnight cultures of *C. albicans* SC5314 were prepared and diluted to an initial inoculum concentration of 1 × 10^5^ CFU/mL in fresh YPD medium. Then 200 μL of the dilution was transferred to each well of 24-well microplate, and IBC was added to each well to yield a final concentration of 1 MIC and incubated at 30 °C. During the culture, 200 μL of the culture were taken at 0, 2, 4, 6, 8, 10, 12, and 24 h, and the absorbance was measured at 600 nm wavelength. Finally, the growth curve was plotted using the concentration of fungal cells determined at OD_600_.

### 2.4 Assessment of cell membrane integrity, metabolic viability, and cell morphology of IBC-treated *C. albicans* SC5314 cells

Both cell membrane integrity and cell metabolic activity of IBC-treated *C. albicans* SC5314 were investigated using confocal laser scanning microscopy (CLSM; Zeiss LSM 880 with Airycan). *C. albicans* SC5314 planktonic cells in logarithmic growth phase per well were treated with different concentrations of 0 MIC, 1 MIC, and 2 MIC IBC for 12 h. The resulting fungal cells were harvested by centrifugation at 4000 × g for 10 min, washed three times with 10 mM PBS (pH 7.0), and resuspended in PBS. For visualization of cell membrane integrity of IBC-treated fungal cells, resuspended cells were thoroughly mixed with a combination of 2.5 μM SYTO 9 and 5 μM PI, and incubated at 25°C for 15-20 min. Subsequently, 10 μL of fungal cells were imaged using CLSM, where the fluorescence emission spectra were measured at excitation/emission wavelengths of 480/500 nm for SYTO 9 and 490/635 nm for PI, respectively. Meanwhile, to examine the metabolic activity of IBC-treated fungal cells, the resuspended cells were mixed with a mixture of 20 μM FUN^®^ 1 and 3 μM CWS and incubated at 25°C for 30-60 min, and then imaged using CLSM with excitation/emission wavelengths of 470/590 nm for FUN^®^ 1 and 488/617 nm for CWS, respectively.

Morphological changes in IBC-treated *C. albicans* SC5314 cells were evaluated by field-emission scanning electron microscopy (FESEM, Nova Nano SEM-450, Hillsboro, USA) (Saikia, K et al., 2017). Fresh fungal cultures at a final concentration of approximately 1 × 10^6^ CFU/mL were seeded into 24-well plates containing the indicated concentrations of IBC (0 MIC, 1 MIC, and 2 MIC) and incubated at 30°C for 12 h. Then the cultures were centrifuged, the supernatant was decanted, the pellets were three times washed with PBS and then pretreated with 2.5% glutaraldehyde at 4°C for 6 h. Subsequently, the fungal cells were dehydrated using the following concentration gradient of cold ethanol with a 10-minute exposure time per change: 30%, 50%, 70%, 80%, 90%, 100%. The resulting cells were subjected to isoamyl acetate at 25°C for 1 h. Finally, the fixed cells were sputtered using ion sputtering, and the morphological changes of all treated and untreated groups was imaged using FESEM.

### 2.5 Investigation of cell membrane potential, mitochondrial membrane potential and ROS in *C. albicans* SC5314 cells

In order to examine the membrane depolarization, fungal cells were cultured, standardized as described above, and treated with various concentrations of IBC (0 MIC, 1/4 MIC, 1/2 MIC, 1 MIC, 3/2 MIC and 2 MIC) at 30°C for 6 h. For examination of cell membrane potential (Veerana et al., 2022), fungal cells were collected by centrifugation at 4000 × g for 10 min, and three times washed with PBS, resuspended in 200 μL of Bis-(1,3-Dibutylbarbituric acid trimethine oxonol) (DiBAC_4_(3), Molecular Probes, Eugene, OR, USA) in PBS. After incubation for 20 min at room temperature, the fluorescence values were determined by measuring the excitation and emission wavelengths at 490 nm and 516 nm using a microliter Multimode Plate Reader (Perkin Elmer, USA). Meanwhile, mitochondrial transmembrane potential was measured using a mitochondrial membrane potential assay kit with JC-10 (Yeasen, China) according to the manufacturer’s instructions. The mean fluorescence intensities of the JC-10 monomers were determined at an emission wavelength of 525 nm using a microliter Multimode Plate Reader (Bao et al., 2021).

To measure the ROS levels in IBC-treated fungal cells, a fluorescent ROS probe 2′,7′-Dichloro-dihydrofluorescein diacetate (DCFH-DA, Beyotime, China) was used. The fungal cells, at least in three biological replicates, were prepared as described above, three times washed with PBS, and mixed with DCFH-DA in PBS for 30-40 min at 37 °C. Subsequently, the stained cells were harvested and resuspended in 1 mL of pre-warmed PBS. Finally, the fluorescence values were examined by measuring the excitation and emission wavelengths of 488 and 525 nm, respectively, using a microliter Multimode Plate Reader (Yu et al., 2021).

### 2.6 Changes in the cell wall and intracellular contents of IBC-treated *C. albicans* SC5314 cells

Changes in cell wall structure and intracellular contents of IBC-treated *C. albicans* SC54314 were examined using transmission electron microscopy (TEM, HITACHI HT7800) (Hu et al., 2019). Fresh fungal cultures with 1 × 10^6^ CFU/mL were seeded into 24-well plates containing 0 MIC and 1 MIC IBC, and incubated at 30°C for 12 h. Then *C. albicans* SC54314 cells were harvested and washed three times with 10 mM PBS. The 2.5% glutaraldehyde solution was subsequently added, fixed at 4°C for 12 h, and washed three times with 10 mM PBS, followed by fixation with 1% osmium acid solution for 2 h at 4°C. Fungal cells were further dehydrated in ethanol at different concentrations (50%, 70%, 80%, 85%, 90%, 95%, and 100%) and treated with acetone for 20 min. The samples were successively treated with 1:3 acetone and epoxy resin as penetrating agents and embedded in an ethoxylated resin. Ultrathin sections were prepared using a diamond slicer and stained for 5-10 min each with a lead citrate solution and a 50% ethanol-saturated solution of UO_2_ acetate. After air-drying, a TEM was used to observe changes in the cell wall and intracellular contents.

### 2.7 Assessment of adhesion of *C. albicans* SC5314 to non-living surfaces

For the adhesion assay (Qian et al., 2022), overnight cultures of *C. albicans* SC5314 were prepared as described above and adjusted with fresh YPD to 10^6^ CFU/mL by serial dilution. Subsequently, 900 μL of cell dilutions were transferred into each well the 24-well plate, where each well contained one sterilized glass coverslips, and the stock solution of IBC was added to each well to yield different concentrations (0 MIC, 1/4 MIC, 1/2 MIC and 1 MIC). The 24-well plates was incubated without shaking at 30°C. After incubation of 4 h, the fungal cells adhering to the glass coverslips were three times washed gently using 10 mM PBS, and mixed with the mixture of FUN^®^ 1 (20 μM) and CWS (3 μM), followed by an incubation of 45 min at 30°C. Finally, the adhesion of fungal cells was examined using CLSM.

### 2.8 Effect of IBC on biofilm formation by *C. albicans* SC5314 cells

The CV staining, CLSM and FESEM were used to examine the effect of IBC on biofilm formation by *C. albicans* SC5314 cell as described previously (Qian et al., 2022). Briefly, The cell culture condition in the effect of IBC on biofilm formation experiment was basically the same as that in the cell adhesion experiment, except that the culture time was increased to 24 h. The resulting biofilm formed on the glass coverslips was subjected to the CV staining, CLSM and FESEM analysis, respectively. For the examination of the effect of IBC on the biofilm formation, the biofilm obtained was washed three times with 10 mM PBS to remove floating cells and stained with 0.1% (w/v) CV staining. After incubation for 30 min at room temperature, the biofilm was washed three times with 10 mM PBS to remove excess dye and observed under a light microscope at 400 × magnification.

For CLSM analysis, the biofilms formed on the glass slide surface underwent several steps. Firstly, they were washed with sterile PBS and then stained in a solution containing 2.5 μM SYTO 9 and 5 μM PI under dark conditions for 15 min. After staining, the samples were rinsed once with sterile PBS to eliminate unbound stains and allowed to air dry. Subsequently, the examination of biofilms was conducted using CLSM. Similarly, for FESEM analysis, the biofilms formed on the glass slide surface subjected to a series of steps. Initially, the biofilms were rinsed with sterile PBS, followed by pre-fixation with 2.5% glutaraldehyde at 4°C for 4 h. Subsequently, the pre-fixed biofilms were washed once again with sterile PBS. The ensuing process involved sequential dehydration using escalating concentrations of ethanol (30%, 50%, 70%, 80%, 90%, and 100%), followed by exposure to isoamyl acetate at 25°C for 1 h. Lastly, the treated biofilms underwent sputter-coating using an ion sputter and were examined using FESEM. Furthermore, for biofilm quantitative examination, the biofilm biomass was determined using the CV assay as described previously.

Next, the inhibition of biofilm formation was evaluated using the previously described method (Haney et al., 2018). Briefly, 900 μL of a fungal suspension, prepared by diluting an overnight culture into YPD medium, was added to the interior wells of a 96-well microplate containing IBC at the final desired concentration. The plates were incubated overnight at 30°C to allow for fungal growth and biofilm formation. Subsequently, the planktonic cells and spent medium were discarded, and the adhered biomass underwent three rinses with distilled water. Following this, the biomass was stained with a 0.1% CV solution for 25 min, and rinsed three times with distilled PBS to eliminate unbound dye. The bound CV dye was then resuspended in 70% ethanol with gentle mixing, and the OD_570_ was recorded using the same sample plate. The biofilm inhibition was quantified relative to the biofilm grown in the absence of IBC (defined as 100% biofilm formation). The results, obtained from at least three distinct biological replicates, were averaged.

### 2.9 Effect of IBC on mature biofilms formed by *C. albicans* SC5314 cells

Next, the CV staining, CLSM, and FESEM were used to assess the dispersal and killing effects of IBC on mature biofilms (Qian et al., 2022). For the mature biofilms growth, *C. albicans* SC5314 cultures prepared as illustrated above were incubated in a 24-well plate at 30°C, in which the glass coverslips were pre-added to form biofilms. After a culture of 48 h, each well of the plate was treated with IBC at a final concentration of 0 MIC, 2 MIC, 4 MIC and 8 MIC for 4 h at 30°C, followed by washing gently twice using 10 mM PBS to remove floating cells. Subsequently, the resulting samples were examined using light microscopy and CLSM, and the CLSM examination was conducted, in which a mixture of SYTO 9 and PI was added to each well to stain mature biofilms, as described above. Additionally, the mature biofilms treated with or without IBC were subjected to FESEM analysis in accordance with the description provided in section 2.8.

Next, the dispersion of mature biofilms was evaluated using the CV assay. Briefly, 900 μL of the fresh fungal suspension (approximately 10^6^ CFU/mL) was transferred into each well of a 96-well microplate, and the plate was incubated at 30°C for 48 h. Following this, IBC was introduced into each well to achieve distinct final concentrations (0 MIC, 2 MIC, 4 MIC, and 8 MIC) for an additional 4 h of culture at 30°C. Then, the quantitative analysis of the biofilm biomass was conducted in accordance with section 2.8. The dispersion of mature biofilms was quantified relative to the mature biofilm grown in the absence of IBC (defined as 100% mature biofilm). The results, obtained from at least three distinct biological replicates, were averaged.

### 2.10 Annexin -FITC/PI double staining of apoptosis

To identify apoptotic and necrotic cells (Broekaert et al., 2016), an annexin V-FITC/PI double staining kit was employed. After exposure to various concentrations of IBC (0 MIC, 1 MIC, and 2 MIC) in each well of a 6-well plate for 6 h, the cells were washed twice in PBS and resuspended in annexin binding buffer. The resuspension was subjected to annexin V-FITC and PI as per the manufacturer’s instructions at 30°C for 30 min in the dark. After staining, the cells were washed with annexin binding buffer and imaged using CLSM. Annexin V−positive and PI−negative (annexin V+/PI−) cells are considered as early apoptotic cells, whereas the double positive cells (annexin V+/PI+) are classified as late apoptotic cells or necrotic cells. Double-negative cells (annexin V−/PI−) were classified as viable cells. The annexin V-FITC fluorescence was measured at excitation/emission wavelengths of 488/525 nm. The group without IBC treatment was used as a negative control.

### 2.11 Autophage assay

For visualization of the autophagosome in IBC-treated fungal cells, fresh fungal cells obtained above were diluted to approximate density of 1 × 10^6^ CFU/mL, and then treated with 0 MIC, 1/2 MIC, and 1 MIC for 6 h. Subsequently, it was incubated with a 100 μM monodasylcadaverine solution at 30°C for 30 min, followed by washing with PBS to remove excess dye. Then fungal cells were analyzed using a CLSM with excitation and emission wavelengths of 335 nm and 512 nm (Lőrincz et al., 2017). In addition, 3-methyladenine (3-MA), a PI3K inhibitor, was used to further explore this mechanism (Shi et al., 2020). Briefly, 1.5 mL fresh fungal cells with a density of 1×10^6^ CFU/mL were added to each well of a 24-well plate and treated with 10 mM 3-MA for 1 h. After treatment, the fungal cells were harvested, washed with fresh YPD, and resuspended in YPD. The resulting resuspensions were treated with various final concentrations of IBC (0 MIC, 1 MIC and 2 MIC) at 30°C for 0.5, 1 and 2 h, respectively. For the cell viability-based detection of autophagy, the cells were harvested following incubation. Then cells was diluted and plated onto YPD at 30°C for another incubation of 24 h in triplicate. Finally, the number of viable cells in the treated samples was calculated by counting the number of live cells.

### 2.12 RNA sequencing and data analysis

RNA-seq was used to evaluate the changes in gene expression in *C. albicans* SC5314 cells in the absence (Ca-1, Ca-2 and Ca-3) and presence (I-Ca-1, I-Ca-2 and I-Ca-3) of IBC. *C. albicans* SC5314 cells were treated with 0 MIC and 1 MIC for 24 h, collected by centrifugation, and gently washed thrice with 10 mM PBS. The samples were entrusted to Shanghai Meiji Biotechnology Co., Ltd. for subsequent processing, including sample preprocessing, RNA-seq analysis, information extraction, data preprocessing, module identification, and deferentially expressed genes (DEGs) analysis. Briefly, total RNA was extracted from independent biological replicates using the RNeasy Plus Mini Kit (Qiagen, Hilden, Germany), according to the manufacturer’s instructions, with an additional DNase digestion step using the RNase-Free DNase Set (Qiagen) to remove the remaining genomic DNA. The concentration and purity of the total RNA were determined using a Nanodrop 2000, followed by RNA integrity number examination using an Agilent 2100. Sequencing was performed using the Illumina NovaSeq 6000 platform, and the raw sequencing data were compared with *C. albicans* genome sequence using the BioConductor package DESeq2 in R 1.16.1 for DEGs analysis between the treated and untreated samples. The DEGs were annotated using the Kyoto Encyclopedia of Genes and Genomes (KEGG) database. KEGG enrichment was carried out using the BioConductor software CLUSTER Profiler 3.4.4 in the R 1.16.1 package to explore the function of the identified DEGs (Yu et al., 2012). and the statistical enrichment of DEGS in the KEGG database was used for pathway enrichment (Kanehisa et al., 2019).

### 2.13 Real-time quantitative reverse transcription PCR

Total RNA was extracted using the TaKaRa MiniBEST Universal RNA Extraction Kit. RT-qPCR assays were performed using the Real-Time PCR Detection System (Bio-Rad) and SYBR Green (Roche) according to the manufacturer’s protocol. A quantitative method (2^-ΔΔCT^) was used to detect changes in expression following the treatment of cells with IBC. All primers were synthesized by Sangon Biotech (Shanghai) Co., Ltd., and all oligonucleotide sequences are shown in **Supplementary Table 1**.

### 2.14 Potential interactions between IBC and target proteins

The PDB database (http://www.rcsb.org/) was used to download the structures of the receptor proteins ATG8, ATG11, ERG11, ERG3, HSP90, and AIF1. Target proteins were removed from water molecules using PyMOL 2.3.0 software. The IBC structure file was downloaded from PubChem (https://pubchem.ncbi.nlm.nih.gov/). Molecular mechanics calculations of the optimal conformation of IBC were performed using the Chem3D software, resulting in an energy-minimized optimal conformation of IBC. Next, the target proteins and IBC were hydrogenated using AutoDock Tools 1.5.6, and determined to be twistable bonds. POCASA was used to predict protein activity pocket. The target proteins and IBC were then docked in a molecular simulation using AutoDock Vina v.1.2.0. The docking algorithm was a Lamarckian genetic algorithm, and the docking method was semi-flexible (Agu et al., 2023).

### 2.15 Hyphal growth analysis

The effect of IBC on hyphal formation in *C. albicans* was examined as described previously (Qian et al., 2022). Briefly, fresh fungal cells prepared above were cultured and diluted to a concentration of 1 × 10^6^ CFU/mL using RPMI 1640 containing 1% fetal bovine serum (FBS) (Thermo Fischer, Belgium). Then 900 μL of fungal cell dilution were transferred into each well of a 24-well flat-bottom plate, followed by the addition of 100 μL of IBC solution to yield various final concentrations (0 MIC, 1/4 MIC, 1/2 MIC and 1 MIC). Subsequently, the mixtures were incubated for 24 h at 30°C in a 5% CO_2_ incubator, and yeast cells and/or hyphae were evaluated microscopically.

### 2.16 Lifespan assay of *C. elegans*

The toxic effect of IBC on *C. elegans* lifespan was evaluated according to a previously described method with slight modifications (Apfeld and Kenyon, 1999). Lifespan measurements were performed in each well of a 24-well plate supplemented with liquid media. Briefly, *C. elegans* worms were cultured on nematode growth medium (NGM) plates seeded with *E. coli* OP50, synchronized until they reached the young adult stage, and washed twice with M9 buffer. Thirty synchronized worms were transferred into each well of a 24-well plate supplemented with various final concentrations of IBC (0 MIC, 1 MIC, 2 MIC, 4 MIC and 8 MIC), along with the addition of 75 μM 5-Fluoro-2’-deoxyuridine (FUDR) in each well, to prevent their progeny from growth unless stated otherwise. The plate was sealed with Breathe-EasyTM membranes, and cultured for 15 days at 22°C. Worm survival was examined using a dissecting microscope and recorded every other day to analyze the survival rate. Worms were considered dead if there was no movement when treated with mechanical stimuli.

In addition, to evaluate the antifungal activities of IBC in *C. albicans* SC5314-infected *C. elegans* model system, an infection assay was performed as previously reported. Briefly, synchronized worms prepared above were washed with M9 buffer, and transferred into *C. albicans* SC5314 lawns that cultured for 48 h at 30°C on YPD, and co-inoculated for another 6 h. Then, thirty *C. albicans* SC5314-infected worms were gently washed twice with M9 buffer, transferred into NGM liquid medium containing IBC at different concentration (0 MIC, 1 MIC and 2 MIC), and incubated for 15 days at 22°C. The survival of the worms was assessed daily using a dissecting microscope, and the survival rates were analyzed. All experiments were performed in triplicates.

### 2.17 Statistical analysis

All data are expressed as the mean ±  standard error of the mean (SEM), and represent at least 3 independent experiments. Statistical differences between groups were analyzed using the GraphPad Prism software. Data were considered statistically significant at *p* < 0.05.

## 3 Results

### 3.1 The MIC and MFC_90_ of IBC against *C. albicans* SC5314

The result showed that MIC and MFC_90_ of IBC against *C. albicans* SC5314 were 8 μg/mL and 16 μg/mL, respectively. The effect of IBC on the growth kinetics of fungal cells was plotted by measuring the OD_600_ at different time points. As presented in **Supplementary Figure 1**, compared with the untreated group, IBC at 1 MIC showed a significant inhibitory effect on the growth of *C. albicans* SC5314 over a 24-h period.

### 3.2 The effect of IBC on cell membrane integrity, metabolic activity, and cell morphology of *C. albicans* SC5314

**Figure 1:**
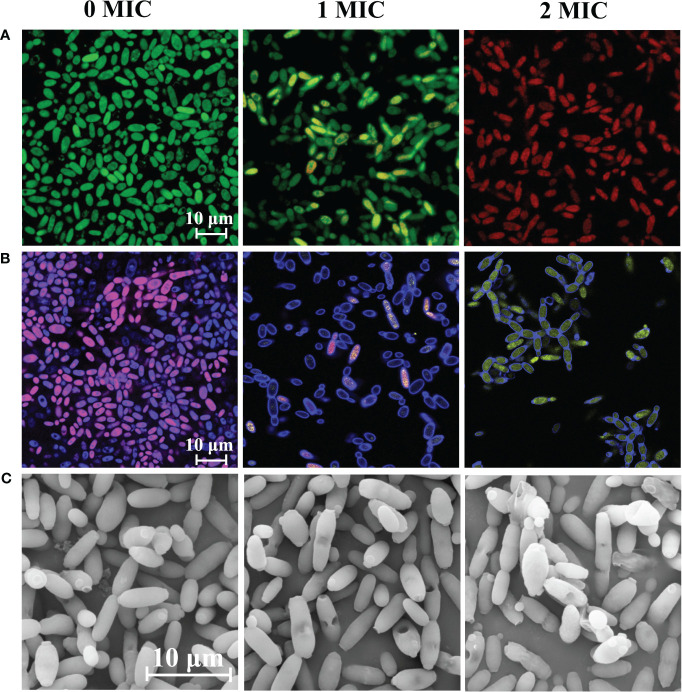
Effect of IBC on the cell membrane integrity **(A)**, metabolic activity **(B)** and cell morphology **(C)** of *C albicans* SC5314. CLSM images of *C albicans* cells treated with 0 MIC, 1 MIC and 2 MIC of IBC using SYTO9/PI **(A)**, and FUN^®^ 1/Calcofluor^®^ White M2R **(B)** double dyes, respectively. *C albicans* SC5314 exposed to various concentrations of IBC at 30°C for 6 h was evaluated using FESEM. The scale bar for CLSM and FESEM images was 10 μm.

SYTO 9 and PI were used to qualitatively evaluate cell membrane integrity. Fungal cells with intact cell membranes emitted green fluorescence upon staining with SYTO 9, whereas fungal cells with damaged membranes emitted red fluorescence upon staining with PI. As shown in **Figure 1A**, there was a significant difference in the integrity of cell membranes between the untreated group and those treated with different concentrations of IBC. The ratio of red to green fluorescence increased with increasing IBC concentration, and almost all fungal cells exhibited red fluorescence in the 2 MIC IBC-treated group, while fungal cells in the untreated group showed clear bright green fluorescence.

FUN^®^, a membrane-permeate nonfluorescent precursor converted by the activity of intracellular enzymes to a fluorescent product, can distinguish among metabolically active, metabolically weakened, and dead cells. As shown in **Figure 1B**, it was almost impossible to observe the transition from red to yellow fluorescence in the untreated group. In contrast, after treatment with 1 MIC or 2 MIC of IBC, the proportion of red fluorescence prominently decreased with the increase in IBC concentrations, and red fluorescence was almost invisible in the 2 MIC IBC-treated group, accompanied by a distinct increase in dark green-yellow or light yellow fluorescence. Therefore, the metabolic activity of *C. albicans* SC5314 cells decreases with increasing IBC concentrations.

Moreover, **Figure 1C** shows that *C. albicans* SC5314 exhibited significant shrinkage or even damage to the surface of fungal cells after treatment with different concentrations of IBC. Notably, when exposed to 2 MIC IBC, extensive damage to the morphology of fungal cells was observed, resulting in the release of intracellular components, whereas the cell morphology of the untreated group had a clear structure and a smooth surface.

### 3.3 IBC reduced the levels of mitochondrial membrane potential and ROS production

**Figure 2:**
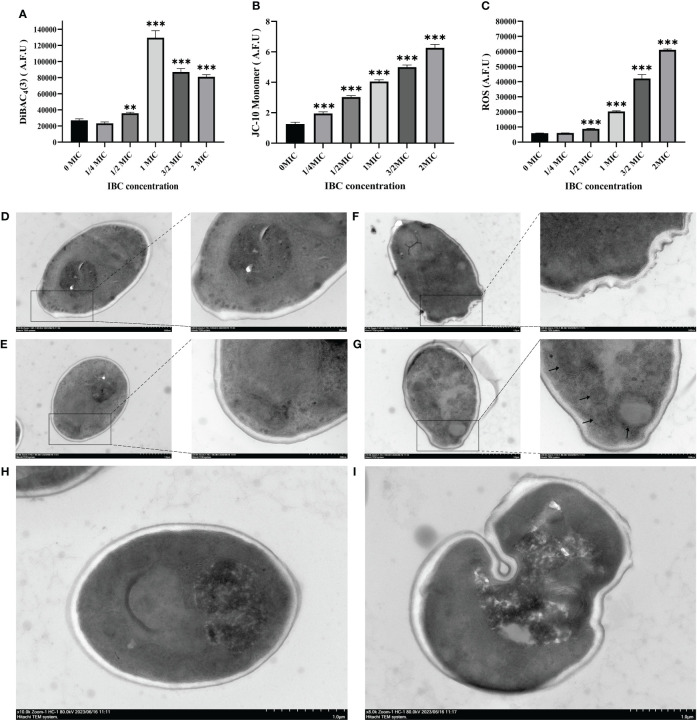
Quantitative analysis of plasma membrane potential **(A)** using DiBAC_4_(3), mitochondrial membrane potential **(B)** using JC-10, and intracellular ROS contents **(C)** using DCFH-DA in IBC-treated *C albicans* SC5314 cells. TEM micrograph of *C albicans* SC5314 cells treated with or without 1 MIC IBC. D-H, approximately 10^6^ fungal cells were co-incubated in the absence **(D, E)** and presence **(F-I)** of IBC at 1 MIC for 24 h. Scale bar was 1 μM for the original images, and 500 nm for the extended images, respectively. AFU, arbitrary fluorescence units. The black arrow indicates the presence of autophagosomes. Bars represent the standard deviation (n = 3). *p < 0.05; **p < 0.01; ***p < 0.001.

The effect of IBC on the plasma membrane of *C. albicans* SC5314 was evaluated by measuring the fluorescence intensity of DiBAC_4_(3), a lipophilic anionic fluorescent dye that is used to quantitatively measure changes in the plasma membrane potential. As shown in **Figure 2A**, there was no significant difference in the fluorescence levels when *C. albicans* SC5314 was treated with the concentration of 1/4 MIC IBC or less. In contrast, after treatment with a concentration of 1/2 MIC or higher, the fluorescence intensity in the treated group showed a significant increase compared to that in the untreated group, indicating that the plasma membrane potential of fungal cells exhibited significant depolarization. Taken together, IBC damages plasma membrane integrity and alters plasma membrane potential in a dose-dependent manner.

Additionally, studies have shown that mitochondria are highly dynamic organelles involved in cellular homeostasis, energy metabolism, ATP production, and protein synthesis. The disruption of MMP and ROS generation is often associated with cell death. First, the effect of IBC on the MMP of *C. albicans* SC5314 was determined by measuring the fluorescence intensities of JC-10 dyes. **Figure 2B** shows that fungal cells treated with IBC at a concentration of 1/4 MIC or higher exhibited an elevation in MMP compared with control cells. This observation was supported by an increase in the green fluorescence intensity of the JC-10 monomer between the 1/4 MIC or higher IBC-treated groups and the untreated group using JC-10 dyes, thereby indicating a dose-dependent effect on MMP (**Figure 2B**). ROS production in *C. albicans* SC5314 cells exposed to IBC was determined by measuring the fluorescence levels of ROS-specific dyes. Treatment with IBC enhanced ROS production in a concentration-dependent manner (**Figure 2C**), and there was a statistically significant difference in the fluorescence intensity between the 1/2 MIC or higher IBC-treated groups and the untreated group (*p* < 0.001).

### 3.4 The effect of IBC on *C. albicans* SC5314 on cell wall and intracellular contents

TEM was used to observe changes in the cell wall and autophagy of *C. albicans* SC5314 exposed to IBC. As shown in **Figures 2D, E**, after the fungal cells were cut into either cross or longitudinal sections, the overall morphology of the untreated fungal cells with intact intracellular components was clearly observed. The cell walls of the fungal cells were intact and smooth, and the mitochondria were intact. In contrast, after treatment with IBC at 1 MIC, a space between the cell wall and the plasma membrane was observed in IBC-treated fungal cells, and the cell wall was thinner than the untreated cells (**Figures 2F, I**). Additionally, it was observed that after the addition of IBC to the media, autophagosome accumulation appeared within the vacuoles of 1 MIC IBC-treated fungal cells (**Figure 2G**). Notably, autolysosomes were observed after treatment with 1 MIC of IBC (**Figures 2H, I**), indicating the potential occurrence of the lysosome-autophagosome contact and fusion.

### 3.5 The inhibitory effect of IBC on cell adhesion of *C. albicans* SC5314 to surfaces

**Figure 3:**
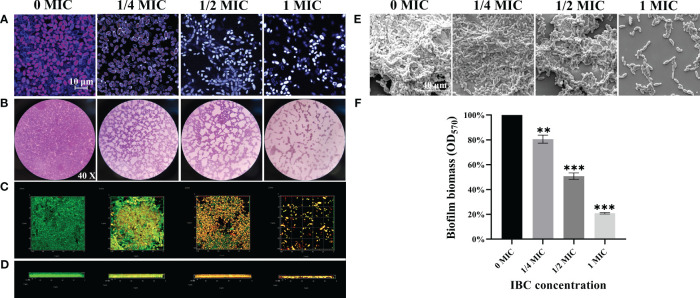
**(A)**. The anti-adhesion ability of IBC against *C albicans* SC5314 to surfaces using FUN^®^ 1/Calcofluor^®^ White M2R double dyes combined with CLSM. **(B-E)**. The antibiofilm effect of IBC on *C albicans* SC5314 was evaluated using CV staining (**B**, objective, × 40), CLSM (**C**, plane images; **D**, three-dimensional images) and FESEM **(E)**. The inhibitory effect of IBC on biofilm formation was quantitatively assessed using CV staining **(F)**. Bars represent the standard deviation (n = 3). Scale bar was 10 μm for CLSM and 40 μm for FESEM, respectively. **p < 0.01; ***p < 0.001.

Next, we evaluated the anti-adhesion ability of IBC against *C. albicans* SC5314 cells. FUN ^®^ and CWS dyes were used using CLSM. **Figure 3A** shows that in the absence of IBC, a large number of fungal cells adhered to the glass coverslips and emitted distinct orange-red fluorescence owing to their excellent metabolic activity. In contrast, when treated with IBC at 1/2 MIC, a significant reduction in the number of adherent cells was observed. Additionally, 1/2 MIC-treated adhered cells emitted grey/white fluorescence because of their low metabolic activity or metabolic inactivity. Increasing the IBC dose resulted in a more pronounced anti-adhesion effect. The preliminary study show that IBC has sufficient anti-adhesion efficacy at 1/2 MIC or higher, in addition to reducing cell metabolic activity results.

### 3.6 Effect of IBC on biofilm formation of *C. albicans* SC5314

The antibiofilm effect of IBC on *C. albicans* SC5314 was evaluated using the CV staining, CLSM, and FESEM. As shown in **Figures 3B-E**, bright purple patches on the images of the untreated group were observed by the CV staining (**Figure 3B**), whereas in the untreated group, fungal cells produced thick and extensive biofilms on the glass coverslips by CLSM (**Figures 3C, D**) and FESEM (**Figure 3E**) analyses. In contrast, the biofilm biomass decreased with an increase in IBC concentration. Mottled purple spots on the images for the 1/2 MIC IBC-treated group were observed by light microscope (**Figure 3C**), and the thickness and density of biofilms were reduced by FESEM analysis (**Figure 3E**). Surprisingly, in the presence of 1/2 MIC IBC, the cell membranes of a small minority of biofilms were damaged, as evidenced by the red fluorescence inside the biofilms observed by CLSM (**Figures 3C, D**). Additionally, in the 1/2 MIC and 1 MIC IBC-treated groups, the biofilm biomass was diminished by 49.2% ± 5.1% and 79.1% ± 3%, respectively, compared to the control group (**Figure 3F**). Collectively, these results indicated that IBC at 1/2 MIC effectively inhibit biofilm formation and compromised biofilm cells of *C. albicans* SC5314.

### 3.7 Ability of IBC to disperse and kill mature biofilms formed by *C. albicans* SC5314

**Figure 4:**
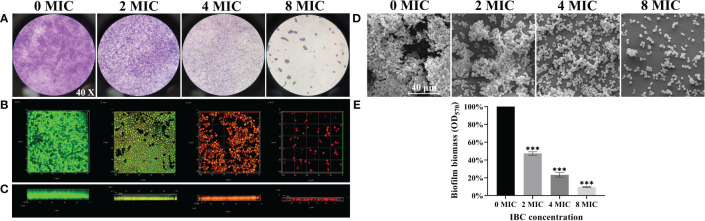
Investigation of the ability of IBC to disperse and kill 48-h mature biofilms by *C. albicans* SC5314. The dispersal and killing effect of IBC on 48-h mature biofilms was evaluated using CV staining (**A**, objective, × 40), CLSM (**B**, plane images; **C**, three-dimensional images) and FESEM **(D)**. The dispersal effect of IBC on preformed biofilms was quantitatively assessed using CV staining **(E)**. Bars represent the standard deviation (n = 3). Scale bar was 10 μm for CLSM and 40 μm for FESEM, respectively. ***p < 0.001.

To investigate the ability of IBC to disperse and kill 48-h mature biofilms, a combination of light microscope, CLSM, and FESEM analyses was employed. Different dimensions were used to explore the dispersal and killing abilities of the IBCs in mature biofilms. As shown in **Figure 4**, mature biofilms without IBC treatment were characterized by the appearance of cell clusters and thickness with a typical multilayer biofilm structure. The addition of IBC post-inoculation resulted in a significant decrease in the total biofilm biomass, as indicated by decreased CV staining in a dose-dependent manner (**Figure 4A**). In contrast, the biofilm cells emitted bright green fluorescence owing to their high survival (**Figures 4B, C**). Surprisingly, the highest biofilm-dispersing activity was achieved at 8 MIC under the conditions tested, and the biomass and metabolic activity of mature biofilms were reduced significantly, as evidenced by the remarkable decline in CV staining intensity (**Figure 4A**) and green fluorescence (**Figures 4B, C**). In addition, IBC at 8 MIC completely destroyed the three-dimensional structure of the biofilm, with the appearance of a few scattered cell aggregates (**Figure 4D**). In the 4 MIC and 8 MIC IBC-treated groups, the mature biofilms were dispersed by 76.7% ± 10.4% and 90.3% ± 6.7%, respectively, in comparison to the control group (**Figure 4E**). These results indicate that IBC could effectively disperse mature biofilms and kill biofilm cells of *C. albicans* SC5314.

### 3.8 Involvement of apoptosis and autophagy in IBC-induced *C. albicans* SC5314 cell death

**Figure 5:**
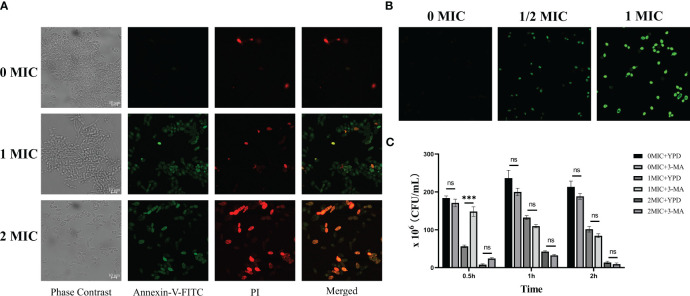
**(A)**. Verification of apoptotic and necrotic cell death using standard methods. CLSM analysis of cells were stained using annexin V-FITC/PI dyes after exposure to different concentrations of IBC. **(B)**. CLSM analysis of cells were stained using monodansylcadaverine dyes. IBC treatment induced the formation of autophagosome in *C albicans* SC5314 cells. **(C)**. 3-Methyladenine (3-MA) caused reversal of 1MIC or 2 MIC IBC-induced autophagy of *C albicans* SC5314 cells at a relatively high level at 0.5 h, but had no effects on the reversal at 1 h and 2 h. Representative results of three repeats were shown, and error bars represented standard errors of the means (n=3). Asterisks indicate significant difference: ns: no significance, ***p < 0.001.

Next, the involvement of apoptosis and autophagy in the IBC-mediated *C. albicans* cell death mechanism was examined. In apoptosis pathway, the IBC-treated *C. albicans* cell examined by CLSM combined with annexin V-FITC/PI double staining. As displayed in **Figure 5A**, consistent with the cell viability assay, early apoptotic (annexin V+/PI−) cells were observed in the presence of 1MIC IBC. In contrast, the number of late apoptotic cells or necrotic cells (annexin V+/PI+) increased markedly when exposed to 2 MIC IBC compared to that of the control group with healthy viable (annexin V−/PI−) cells, indicating that IBC-induced apoptosis in *C. albicans* in a concentration-dependent manner. In addition to inducing cell apoptosis, IBC can also induce autophagy in *C. albicans* according to the TEM analysis.

Monodansylcadaverine is an autofluorescent amine that specifically stains autophagosomes in mammalian cells (Biederbick et al., 1995). As shown in **Figure 5B**, strong monodansylcadaverine staining was observed within *C. albicans* cells after treatment with 1 MIC or 2 MIC IBC, and the fluorescence intensity significantly increased with an increase in IBC concentration. Moreover, after treatment with 1 MIC IBC, *C. albicans* cells exhibited prominent fluorescent vesicular structures. These results indicate that IBC treatment induces the formation of autophagosome in *C. albicans* cells.

In addition, to analyze the potential mechanism of action, we assessed the effect of exposure to 3-MA, a PI3K pathway inhibitor, on autophagy in IBC-treated *C. albicans* cells by simultaneous administration of various concentrations of IBC ranging from 0 MIC to 2 MIC. At 0.5, 1, and 2 h, treated fungal cell samples were collected for examination. As shown in **Figure 5C**, 3-MA caused reversal of 1 MIC or 2 MIC IBC-induced autophagy of *C. albicans* cells at a relatively high level at 0.5 h. However, with the prolongation of the time of IBC action, the reversal of autophagy caused by 3-MA disappeared at both 1 MIC and 2 MIC IBC treatment concentrations at 1 and 2 h in a time-dependent manner. Taken together, these results indicate that IBC induces autophagy by inhibiting the formation of other complexes, in addition to targeting the classical PI3K complex.

### 3.9 Transcriptomic analysis of the effect of IBC on *C. albicans* SC5314

**Figure 6:**
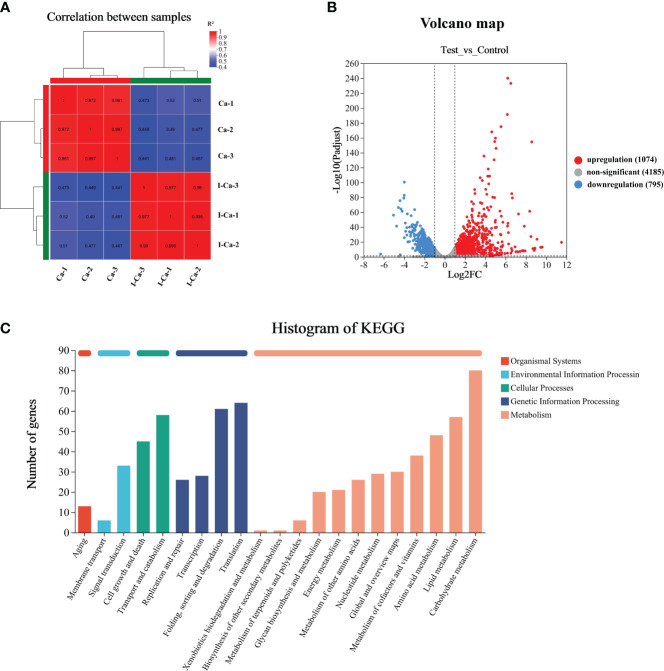
RNA-seq correlation and differential gene expression analysis of untreated and IBC-treated *C albicans* SC5314 cells. RNA-seq analysis was carried out on total RNA extracted from three replicate samples for each biological group. The Pearson R2 correlation heat map of gene expression levels between six samples was displayed in **(A)**. The volcano map of mRNA expression in untreated versus IBC-treated *C albicans* SC5314 cells **(B)**. KEGG Pathway analyses was performed to identify biological processes significantly enriched with differentially expressed genes **(C)**.

**Figure 7:**
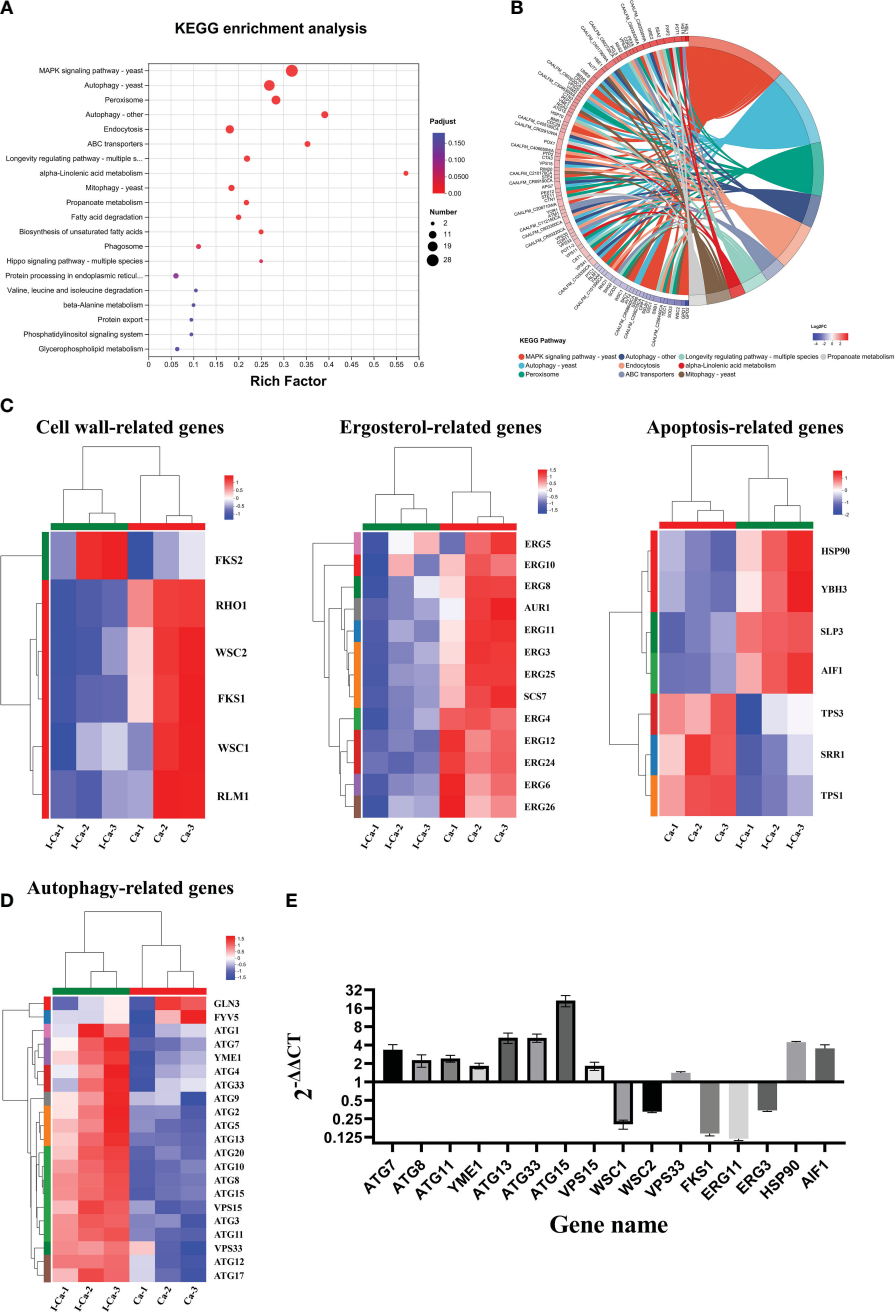
**(A)**. KEGG pathway analysis of differentially expressed genes (DEGs) was performed to identify the most significantly enriched pathways at a threshold Padjust of ≤ 0.05. The size of the dots represented the number of DEGs enriched in the pathway. **(B)**. Chord diagram showing the top 10 enriched KEGG clusters was plotted. In each chord diagram, enriched KEGG clusters were shown on the right and genes contributing to enrichment were shown on the left. Nodes of different colors represent the differential genes enriched in each KEGG pathway. The blue and red nodes represented downregulated and upregulated, respectively. **(C, D)**. Heatmaps showing the expression of key genes associated with cell wall biosynthesis, ergosterol biosynthesis, apoptosis **(C)**, and autophagy **(D)** pathway. Fold changes, compared to the control cells, of down-regulation (blue) and upregulation (red) were shown on a log2 scale for untreated and treated group. **(E)**. Validation of sixteen genes by quantitative reverse transcription polymerase chain reaction. The bars represented the average relative change in RNA abundance of the indicated genes, and the error bars represent the standard deviation (n = 3 in each sample).

To investigate the mechanism by which IBC affects *C. albicans* SC5314 growth at the molecular level, we analyzed the entire transcriptome of *C. albicans* SC5314 treated with IBC using RNA sequencing. As shown in **Figure 6A**, good reproducibility in both the treated and control groups and a significant difference between the treated and untreated groups were observed. **Figure 6B** shows that a total of 6054 expressed genes were detected, among which 1869 genes were significantly up- or downregulated (*p* < 0.5), including 1074 upregulated genes and 795 downregulated genes. To provide an overview of the functions and processes linked to the DEGs in the transcriptome, KEGG enrichment analysis was performed (**Figure 6C**). In addition, the expression of typical marker genes and gene families was analyzed. KEGG analysis revealed that DEGs were mainly enriched in five biological processes related to organismal systems: environmental information processing, cellular processes, genetic information processing, and metabolism. KEGG pathway enrichment analysis of all DEGs was performed to identify several major cell death-related pathways. These pathways mainly involved the cell wall, cell membrane, autophagy, and peroxisomal pathways (**Figures 7A, B**). Remarkably, several key genes related to cell wall organization were downregulated, such as *Wsc1*, *Wsc2*, *Rho1*, *Rlm1*, and *Fks1*, whereas *Fks2* was upregulated (**Figure 7C**). Of these, *Wsc1* and *Wsc2*, which belong to the PKC1-MPK1 pathway that maintains cell wall integrity, encode predicted integral membrane proteins (García et al., 2019). *Rho1* functioned in the organization of the actin cytoskeleton, and directly modulated cell wall biosynthesis by activation of β-glucan synthase (Martínez-Rocha et al., 2008). The *Rlm1* gene encoded a transcription factor, one of the putative transcription factors involved in the cell wall integrity, and *C. albicans* Δ/Δ*rlm1* mutants showed typical cell wall weakening phenotypes (Xie et al., 2023). Additionally, Fks1 gene, encoding β-1,3-glucan synthase, is involved in the biosynthesis of β-1,3-glucan, the core component of the fungal cell wall, whereas *Fks2* acted as the negative regulator of *Fks1* (Suwunnakorn et al., 2018; Hu et al., 2023).

In the fungal cell membrane pathway, ergosterol is an indispensable component of membrane lipids that modulate fluidity and maintain the permeability and integrity of the plasma membrane (Guo et al., 2023). In addition, the expression of 11 genes related to ergosterol biosynthesis was significantly downregulated in response to IBC treatment (**Figure 7C**). In particular, eight genes encoding key ergosterol biosynthesis enzymes, namely *Erg3* (Branco et al., 2017), *Erg4* (Han et al., 2023), *Erg5* (Sun et al., 2013), *Erg6* (Oliveira et al., 2020), *Erg11* (Jordá and Puig, 2020), *Erg24* (Li et al., 2021), *Erg25* (Blosser et al., 2014), and *Erg26* (Germann et al., 2005), were significantly downregulated. Of particular interest are *Erg3* and *Erg11* because they are the most important genes in the ergosterol biosynthesis pathway and have key roles in azole drug resistance. In addition, *Erg6*, which is involved in the ergosterol biosynthesis pathway and may act on similar substrates, leading to the formation of several sterol intermediates, was downregulated. Additionally, *Aur1*, which is involved in the biosynthesis of sphingolipids that act as components of cellular membranes, was downregulated (Hameed et al., 2011).

The apoptosis and autophagy pathways seemed to be specifically affected. **Figure 7C** shows the significantly regulated apoptosis-related genes. Of these, four pro-apoptosis-related genes (*Hsp90*, *Ybh3*, *Aif1*, and *Slp3*) (Büttner et al., 2011; Muzaffar and Chattoo, 2017; Conrad et al., 2018; Peng et al., 2022) were significantly upregulated in IBC-treated *C. albicans* SC5314 cells, whereas three apoptosis-related genes (*Srr1*, *Tps1*, and *Tps3*) (Wilson et al., 2007; Mavrianos et al., 2013; Fichtner et al., 2020) were significantly downregulated. Among these, *Hsp90* gene was significantly upregulated. Previous studies showed that compromised *Hsp90* reduces apoptosis in *C. albicans* and that genetic depletion of *Hsp90* attenuates virulence in a mouse model of invasive candidiasis (Dai et al., 2012; Robbins and Cowen, 2023). Similarly, *Aif1*, a mitochondria-localized protein, plays a dual role in the regulation of cell death, and the deletion of *Aif1* attenuates apoptosis (Ma et al., 2016). *Ybh3* functions as a pro-apoptotic regulator and enhances apoptotic phenotypes upon its overexpression (Nam et al., 2022). Additionally, the *C. albicans tps1*Δ mutant, which is deficient in trehalose synthesis, exhibited increased apoptosis rate upon H_2_O_2_ treatment (Lu et al., 2011).

As shown in **Figure 7D**, autophagy-related (Atg) upregulated at almost all steps. In particular, *Atg1*, *Atg13*, and *Atg17*, which encode proteins that act as components of the Atg1 complex (Cai et al., 2022), were upregulated. Similarly, *Atg12*, *Atg5*, and *Atg16*, which encode proteins that act as components of the Atg12-Atg5-Atg16 complex (Walczak and Martens, 2013) and are recruited to the pre-autophagosomal structure (PAS), were upregulated. *Vps15*, which encodes a protein that acts as a component of class III PI3K complex I (PI3K) (Anding and Baehrecke, 2015; Iershov et al., 2019), was also overexpressed. Notably, *Atg8* gene (Shpilka et al., 2011), encoding a core protein involved in autophagy, was significantly upregulated. In addition, *Atg33* gene (Kanki et al., 2011), which is capable of completely inhibiting yeast mitophagy in the post-logarithmic phase, was upregulated.

### 3.10 Validation of RNA-Seq data by RT-qPCR analysis

To further validate the RNA-Seq expression data, the same total RNA samples used for RNA-Seq were examined by RT-qPCR. Sixteen DEGs were selected for RT-qPCR analysis, including three cell wall biosynthesis-related genes (*Wsc1*, *Wsc2*, and *Fks1*), nine autophagy-related genes (six Atg*s*, *Yme1*, *Vps15*, and *Vps33*), and six other key genes (**Figure 7E**). Of these, 13 genes were upregulated and five genes were downregulated in the RNA-seq data. The expression data from RNA-Seq and RT-qPCR were compared, and the RT-qPCR results were strongly consistent with the RNA-Seq data, demonstrating the reliability of the RNA-Seq expression profiles in this study.

### 3.11 Molecular docking

**Figure 8:**
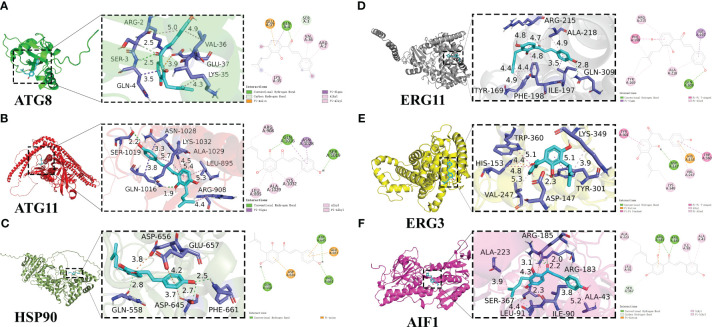
Detailed diagram of molecular docking between IBC and target protiens, including ATG8 **(A)**, ATG11 **(B)**, HSP90 **(C)**, ERG11 **(D)**, ERG3 **(E)**, AIF1 **(F)**. IBC was represented in light blue, and local magnification of the docking sites and 2D diagram of the interactions were shown.

To predict how IBC binds to their protein targets and interacts with amino acids residues from target proteins, six potential target genes for IBC action based on phenotype analysis and transcriptome data, including *Atg8*, *Atg11*, *Hsp90*, *Erg11*, *Erg3*, and *Aif1*, were examined using molecular docking (**Figure 8**). In this study, an IBC/target protein interaction model was developed using molecular docking and molecular dynamics simulations. The binding free energies of the interactions between IBC and the target proteins were calculated using the molecular mechanics generalized Born surface area method. The results show that the binding free energies of interaction between IBC and five proteins were less than -6 Kcal/mol. Among these, the binding energies between ERG3 and ERG11 proteins and IBC was −8.4 Kcal/mol, indicating the strong interaction between IBC with ERG3 and ERG11 proteins. The biphenyl ring structure of IBC forms a π-π stacked structure with amino acid residues from both ERG3 and ERG11 proteins, which is similar to the interaction of sophisticated non-covalent hydrogen bonds. In addition, the biphenyl ring also forms a pi-alkyl structure with amino acid residues from both ERG3 and ERG11 proteins, resulting in the formation of a hydrophobic structure that can effectively increase the interaction between IBC and target proteins.

By analyzing the binding of IBC to both ATG8 and ATG11 proteins, it was found that IBC can not only form hydrogen bonds with amino acid residues from both ATG8 and ATG11 proteins, but also form pi sigma conjugated structures. The binding ability of this conjugated structure is lower than that of hydrogen bonds, but the pi sigma conjugated structure can affect the molecular structure, further intensifying the binding ability of IBC to both ATG8 and ATG11 proteins. In contrast, no strong intermolecular interaction between IBC and HSP90 protein such as hydrogen bonding, were found. Among the six proteins examined, IBC had the highest binding energy with HSP90 protein (−6.4 Kcal/mol). Further analysis of the binding sites of IBC revealed that the effective binding sites between IBC and the six proteins mentioned above were the diphenyl ring structure and the enomethyl site of IBC, indicating that the effective antifungal functional groups of IBC are the diphenyl ring structure and the enomethyl group.

### 3.12 IBC inhibited the hyphal growth of *C. albicans* SC5314

**Figure 9:**
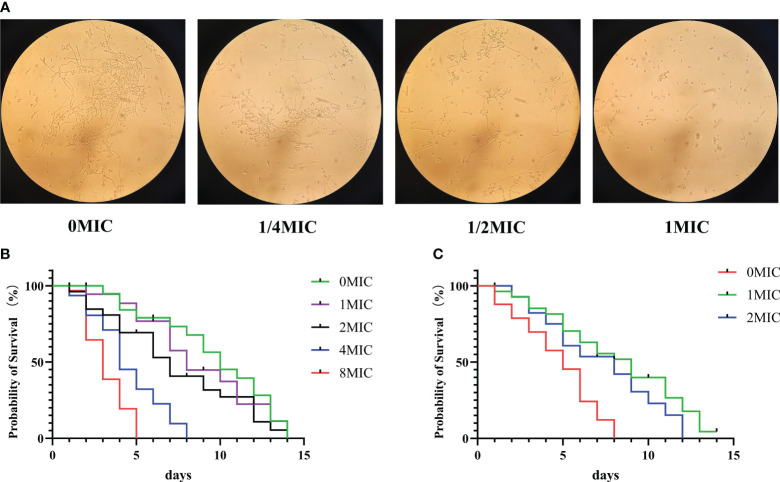
**(A)**. Effect of sub-MIC IBC on the formation of *C albicans* SC5314 hyphae was performed and imaged using light microscopy. **(B)**. Lifespan of *C elegans* worms was determined in the presence of high concentrations of IBC ranging from 0 MIC to 8 MIC, and the survival curve was plotted. **(C)**. Effect of IBC on the lifespan of *C albicans*-infected *C elegans* nematodes was determined, and the survival curve was plotted. Survival of *C* elegans was evaluated in different treatment groups. Values represented the mean survival rates of three biological replicates.

In *C. albicans*, the hyphal morphology is critical for enhancing virulence and pathogenicity because it enables the fungus to attach to and invade the underlying substrate. As shown in **Figure 9A**, in the absence of IBC, the microscopic images showed the formation of extensive hyphae of *C. albicans* in an elongated thread-like form, with *C. albicans* growing as long filamentous hyphae. In contrast, in the presence of IBC, the growth of *C. albicans* in yeast occurred in a concentration-dependent manner. Notably, when treated with 1/2 MIC of IBC, the majority of *C. albicans* cells retained their yeast morphology. In addition, when exposed to 1 MIC of IBC, an almost complete suppression of hyphal growth in *C. albicans* was observed, indicating that IBC can inhibit the hyphal growth of *C. albicans.*

### 3.13 IBC increased the *C. albicans*-infected *C. elegans* lifespan without toxicity

To evaluate the effect of IBC on *C. elegans* lifespan, young worms were exposed to various concentrations of IBC ranging from 0 MIC to 8 MIC for 24 h. As displayed in **Figure 9B**, IBC at the concentrations of 1 MIC, 2 MIC and 4 MIC did not affect the lifespan of worms with the mean lifespan of N_2_ worms of 14.1  ±  0.1 days, 13.3  ±  0.5 days and 8.5  ±  0.3 days, respectively, compared to 14.3  ±  0.3 days of the control group. In contrast, worm lifespan was reduced at a concentration of 8 MIC, with a mean lifespan of 4.1  ±  0.7 days. Additionally, to test the antifungal effect of IBC in *C. albicans*-infected *C. elegans* model, we evaluated the effect of IBC on the lifespan of *C. albicans*-infected worms. As shown in **Figure 9C**, *C. albicans-*infected worms had a mean lifespan of 7.4 ± 0.1 days. By contrast, in the presence of 1 MIC and 2 MIC IBC, *C. albicans-*infected N_2_ worms lived on average for 14.1 ± 0.5 days and 13.1 ± 0.2 days, with a marked extension of the mean lifespan by 104.2% and 85.9% compared with 7.4 ± 0.1 days of the control group, respectively. Taken together, these results indicated that IBC exposure increased *C. albicans*-infected *C. elegans* lifespan proportional to a dose-dependent manner.

### 3.14 Summary of IBC-induced cell death mechanism in *C. albicans* cells

**Figure 10:**
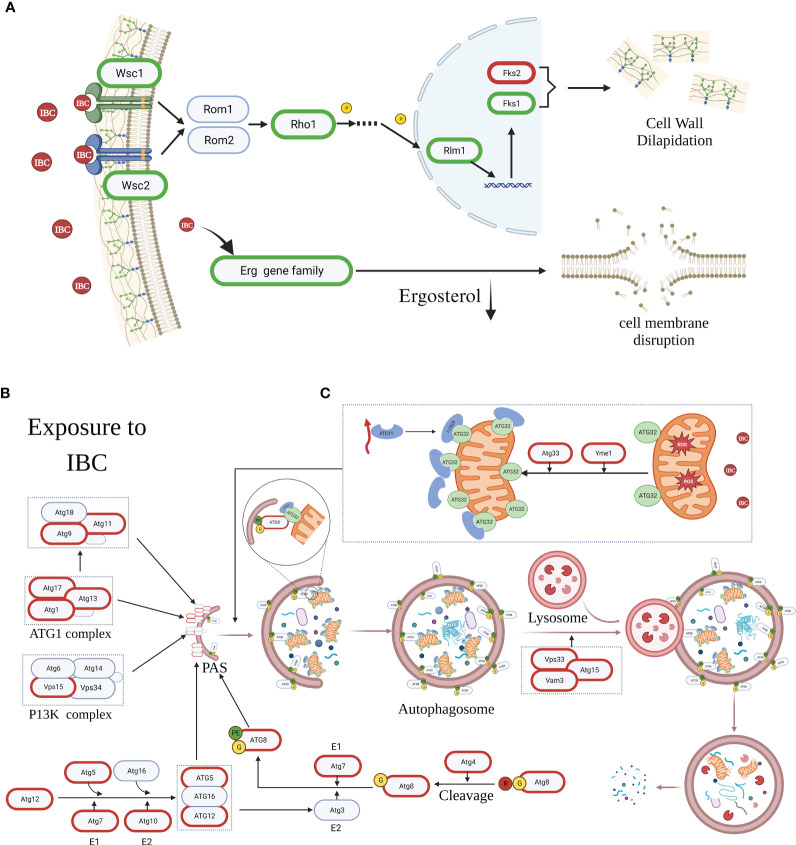
The mechanism diagram of IBC-induced cell death mechanism in *C albicans* cells. **(A)**. IBC exerted antifungal effects through inhibiting the cell wall/membrane synthesis of fungal cell by downregulating expression of key genes such as *Wsc1*, *Wsc2*, *Fks1*, *Rho1* and *Rhm1*, and upregulating the expression of *Fks2* gene. **(B)**. IBC treatment caused autophagy-mediated *C albicans* cell death by upregulating the expression of most of autophagy-related (Atg) genes associated with the formation of Atg1 comlex, Atg5-Atg12-Atg16 complex, and the lipidated protein Atg8-PE, thereby promoting the formation of the pre-autophagosomal structure (PAS). **(C)**. IBC treatment enhanced ROS overproduction and yielded mitochondrial depolarization, and upregulated the expression of *Atg33* gene, which resulted in the Atg32-mediated mitophagy. Genes circled in red and green are significantly upregulated and downregulated, respectively. PE, the lipid phosphatidylethanolamine.

IBC exerts antifungal effects by inhibiting the synthesis of fungal cell walls/membranes and by inducing apoptosis and autophagy in *C. albicans* cells. As shown in **Figure 10A**, in cell wall biosynthesis pathway, there was a significant downregulation in expression of *Wsc1* and *Wsc2* genes acting as the cell wall integrity sensor, and a significant downregulation in expression of *Fks1* and *Rhol* genes, translated into the subunits of β-1,3-glucan synthases, along with a marked upregulation in expression of *Fks2* gene, a negative regulator of *Fks1*, as evidenced by damaged cell walls by TEM analysis. Similarly, the expression of most Erg genes related to ergosterol biosynthesis, such as *Erg3*, *Erg6*, and *Erg11*, was significantly downregulated in IBC-treated *C. albicans* cells, which was consistent with SYTO 9/PI double staining for cell membrane permeability (**Figure 10A**). Similarly, transcriptome and RT-qPCR results showed that the expression of apoptosis-related genes, such as *Hsp90* and *Slp3*, was significantly upregulated. This observation was further supported by the increase in the number of early and late apoptotic cells, as demonstrated by the enhance in the amount of annexin V+/PI− cells and annexin V+/PI+ cells using CLSM analysis. Additionally, the transcriptome and qRT-PCR results showed significant alterations in the expression of most autophagy-related genes associated with the formation of the Atg1 complex and PAS, indicating the involvement of autophagy in IBC-treated *C. albicans* cells (**Figures 10B, C**). In TEM images, autophagosomes were observed in IBC-treated *C. albicans*. In conclusion, the potential mechanisms underlying IBC-induced cell death include the disruption of the integrity of the fungal wall/membrane and the induction of apoptosis and autophagy, thereby resulting in the death of *C. albicans*.

## 4 Discussion

*C. albicans* is a ubiquitous opportunistic fungal pathogen that commonly resides in the mucosa of humans, with a worldwide distribution (Lopes and Lionakis, 2022). Changes in microbial homeostasis due to endogenous and exogenous factors (e.g. host immunity, antibiotics, stress, or resident microbiota) can cause the overgrowth of *C. albicans*, thereby leading to a wide range of opportunistic infections in humans, from superficial to life-threatening systemic infections (Lopes and Lionakis, 2022). It is estimated that *C. albicans* affects more than one billion individuals globally each year, posing a mounting threat to global health. Previous evidences have demonstrated that *C. albicans* biofilms formed on medical devices and host tissues (Nobile and Johnson, 2015; Fan et al., 2022), a closely packed community of cells embedded in an extracellular matrix, make it harder to combat fungal infections owing to increased resistance to conventional antifungals, the host immune system, and other environmental perturbations. *C. albicans* biofilms cause disease through biofilm-based device- or tissue-related healthcare-associated infections, posing a rising global health threat. In addition, *C. albicans* biofilms are a major deposit of persistent fungal cells, which most likely contribute to the resistance and recalcitrance of biofilm infections as well as the clinical recurrence of these infections (Pereira et al., 2021). Further compounding this challenge is the limited number of commonly used antifungals available to combat biofilm-based fungal infections in pipeline (Wall and Lopez-Ribot, 2020). In this context, there is an urgent need to exploit novel and alternative antifungal agents to treat medically unmet fungal infections.

Fungal cell membranes are potential targets for novel antifungal agents because membrane integrity is critical for cellular homeostasis and normal cell viability (Sant et al., 2016). Membrane integrity is the distinctive feature most frequently employed to assess whether fungal cells cultivated *in vitro* are alive or dead (Riss and Moravec, 2004). In this study, the results of CLSM combined with various dyes clearly show that IBC causes the loss of membrane integrity, and reduces the metabolic activities of *C. albicans* SC5314. Similar studies have reported that IBC exhibit antibacterial activity against *Clostridium difficile* and *Bacillus subtilis* by disrupting the cell membrane integrity (Assis et al., 2022; Liu et al., 2022). Together, these findings indicated that IBC effectively inhibited the growth of *C. albicans* SC5314 *in vitro* by altering cellular permeability.

Next, we investigated the antibiofilm activity of IBC against *C. albicans* SC5314. Biofilm formation typically four distinct stages (Sauer et al., 2022). The first key step is the initial surface attachment of single free-floating planktonic cells. Once formed, the mature biofilm enables antifungal treatments indispensable to the fungal cells, thereby making them extremely difficult to treat and eradicate effectively (Zarnowski et al., 2021). Therefore, inhibiting the initial fungal attachment and dispersal of mature biofilms is critical for preventing and combating biofilm-related infections. In this study, the results indicated that sub-inhibitory concentrations of IBC with 4 μg/mL effectively prohibit fungal initial adhesion to surfaces. In addition, IBC at the concentration of 64 μg/mL justified the potent efficacy for the dispersal of 48-h mature biofilms by *C. albicans* SC5314, and damaged cell membrane of dispersed fungal cells. These results indicate that IBC can attack the initial and final stages of biofilm formation and development.

The mechanism of action of IBC against *C. albicans* SC5314 was evaluated using TEM, CLSM and RNA sequencing. TEM images clearly indicated that the cell surface of IBC-treated *C. albicans* SC5314 cells had pits and perforations on the cell surface. Additionally, autophagic vesicles in IBC-treated *C. albicans* SC5314 cells were clearly distinguishable, indicating that IBC effectively suppressed fungal cell growth by inducing autophagy in TEM and CLSM images. RNA sequencing was performed to analyze the potential molecular mechanisms underlying antifungal action. The results of RNA sequencing and RT-qPCR revealed that IBC exerts significant antifungal and antibiofilm activities, mainly by interfering with the cell wall/membrane biosynthesis, and activating apoptosis and autophagy pathways. Interestingly, we also observed significant downregulation of *Wsc1*, *Wsc2*, and *Fks1* expression, leading to the damage of the cell wall. Previous studies showed that in *S. cerevisiae*, genetic studies have displayed that *Wsc1* (Schöppner et al., 2022) and *Wsc2* (Philip and Levin, 2001) are essential for cell wall integrity, thereby conferring resistance against multiple types of cell wall stress, including exposure of cells to the β-1,3-glucan synthase inhibitor caspofungin, the chitin-binding agents Congo red and Calcofluor white, or caffeine (Schöppner et al., 2022). In addition, as previously stated, β-1,3-Glucan is an essential matrix component in the fungal cell wall, and is synthesized by membrane-integrated synthase FKS, thus blocking its biosynthesis is also an important strategy for exploiting novel antifungal drugs (Szymański et al., 2022; Hu et al., 2023). In *C. albicans*, *Fks1* is essential for viability (Lee et al., 2023). Thus, it appears that IBC disrupts cell wall integrity by regulating the expression levels of important cell wall integrity-related genes, such as *Wsc1*, *Wsc2*, and *Fks1* genes.

In addition, when *C. albicans* cells were exposed to IBC, a significant downregulation of most of Erg family genes was observed. Erg family genes have been proposed to be involved in the ergosterol synthesis pathway in the cell plasma membrane (Suwunnakorn et al., 2018). Among these, *Erg11* (Vu and Moye-Rowley, 2022) and *Erg3* (Branco et al., 2017) are the most important genes involved in ergosterol biosynthesis pathway (Lee et al., 2023). Fluconazole, a clinical first-line fungistatic antifungal azole, interferes with ergosterol biosynthesis in *C. albicans* by binding to *Erg11*, and thus leads to the accumulation of toxic sterols. In addition to their essential regulatory role in ergosterol biosynthesis, Erg genes play a key role in regulating biofilm formation and morphological transformation (Borecká-Melkusová et al., 2009). For instance, biofilms generally differ in their sterol content and diversity at phenotypically distinct stages compared with planktonic cells. Several studies have shown a remarkable decrease in ergosterol levels in both the intermediate (12-30 h) and mature stages (31-72 h) of biofilm formation, compared to those in the early stage (0-11 h) of biofilm formation, which is linked to the decreased expression of three ergosterol biosynthetic genes, including *Erg25* (Okamoto et al., 2022), *Erg11*, and *Erg6* (Lv et al., 2016; Jordá and Puig, 2020; Oliveira et al., 2020). These might partially explain the efficacy of IBC against cell membrane integrity and biofilm formation of *C. albicans.*

Our data also showed a strong correlation between the extent of apoptosis following treatment with IBC in *C. albicans* cells, as evidenced by the increase in green fluorescence signals with increasing IBC concentrations. The transcriptome strongly supports the results of these phenotypic studies. Specifically, IBC increased mRNA levels of *Aif1*, and decreased *Srr1* and *Tps1*. *Aif1* is positively related with apoptosis (Li et al., 2020). A previous study showed that the level of apoptosis increased remarkably in the *Srr1*Δ mutant strain of *C. albicans* compared to that in the wild type, suggesting the activation of a mitochondrion-dependent apoptotic cell death pathway in the *Srr1*Δ mutant (Mavrianos et al., 2013). Similarly, the *Tps1*Δ mutant of *C. albicans*, which is deficient in trehalose synthesis, demonstrated enhanced apoptosis (Lu et al., 2011).

In IBC-treated fungal cells, the majority of Atg gene expression was significant upregulation, including *Atg1*, *Atg5*, *Atg8*, *Atg11*, *Atg12*, *Atg13*, *Atg16*, *Atg17*, and *Atg33*. Of these, upregulating the expression of most autophagy-related (Atg) genes associated with the formation of the Atg1 complex, Atg5-Atg12-Atg16 complex, and lipidated protein Atg8-PE promotes the formation of the pre-autophagosomal structure (PAS), thus accelerating the autophagy process in IBC-treated *C. albicans.* In addition, previous studies have shown that the expression of *Atg8*, an autophagy factor, is essential for autophagosome formation, and the upregulation of Atg8 expression can accelerate autophagy in cells (Lystad and Simonsen, 2019). Similarly, ATG11 proteins functions as a protein scaffold and membrane-tether component essential for initiating autophagosome formation in yeast and plays a critical role in organizing and activating selective autophagy-specific phagophore assembly sites by interacting with SAR/cargo complexes (Zientara-Rytter and Subramani, 2020). In addition, the deletion of *Gln3* increased the expression of *Atg8* and *Atg29* (Mao et al., 2013) under growing conditions. Thus, upregulation of *Gln3* after IBC treatment effectively induced fungal autophagy. These results suggested that IBC plays an antifungal role by inducing autophagy-dependent fungal cell death.

## 5 Conclusions

This study explored the antifungal and antibiofilm efficacy of IBC, and the results provide important insights into the potential multifunctional mechanism of action of the fungicidal effect of IBC. Future molecular characterization of these genes and pathways in response to IBC is needed to identify appropriate antifungal drugs with multiple targets.

## Data availability statement

The datasets presented in this study can be found in online repositories. The names of the repository/repositories and accession number(s) can be found below: https://www.ncbi.nlm.nih.gov/bioproject/PRJNA1047273/.

## Author contributions

WQ: Writing – original draft. JL: Writing – original draft. CG: Writing – review & editing. QL: Validation, Writing – review & editing. WY: Supervision, Writing – review & editing. TW: Writing – review & editing. XW: Writing – review & editing. ZW: Writing – review & editing.

## Supplementary material

Supplementary Figure 1 Killing-growth curve analysis of *C. albicans* SC5314 strains in the absence and presence of 1MIC IBC. *C. albicans* SC5314 (3 replicates) was grown in a deep 24-well plate at 30°C. For determining the growth, 200 μL were aspirated every 2 h and monitored as OD_600_ for 24 h using the growth reader under constant shaking. The growth curve was prepared based on the growth of three independent cultures, and the mean ± SD was plotted.
