# Supplementary figures and images for: Isobavachalcone exhibits antifungal and antibiofilm effects against C. albicans by disrupting cell wall/membrane integrity and inducing apoptosis and autophagy
Source: Front Cell Infect Microbiol. 2024 Jan 22;14:1336773. doi: 10.3389/fcimb.2024.1336773 (PMC10845358; doi:10.3389/fcimb.2024.1336773)

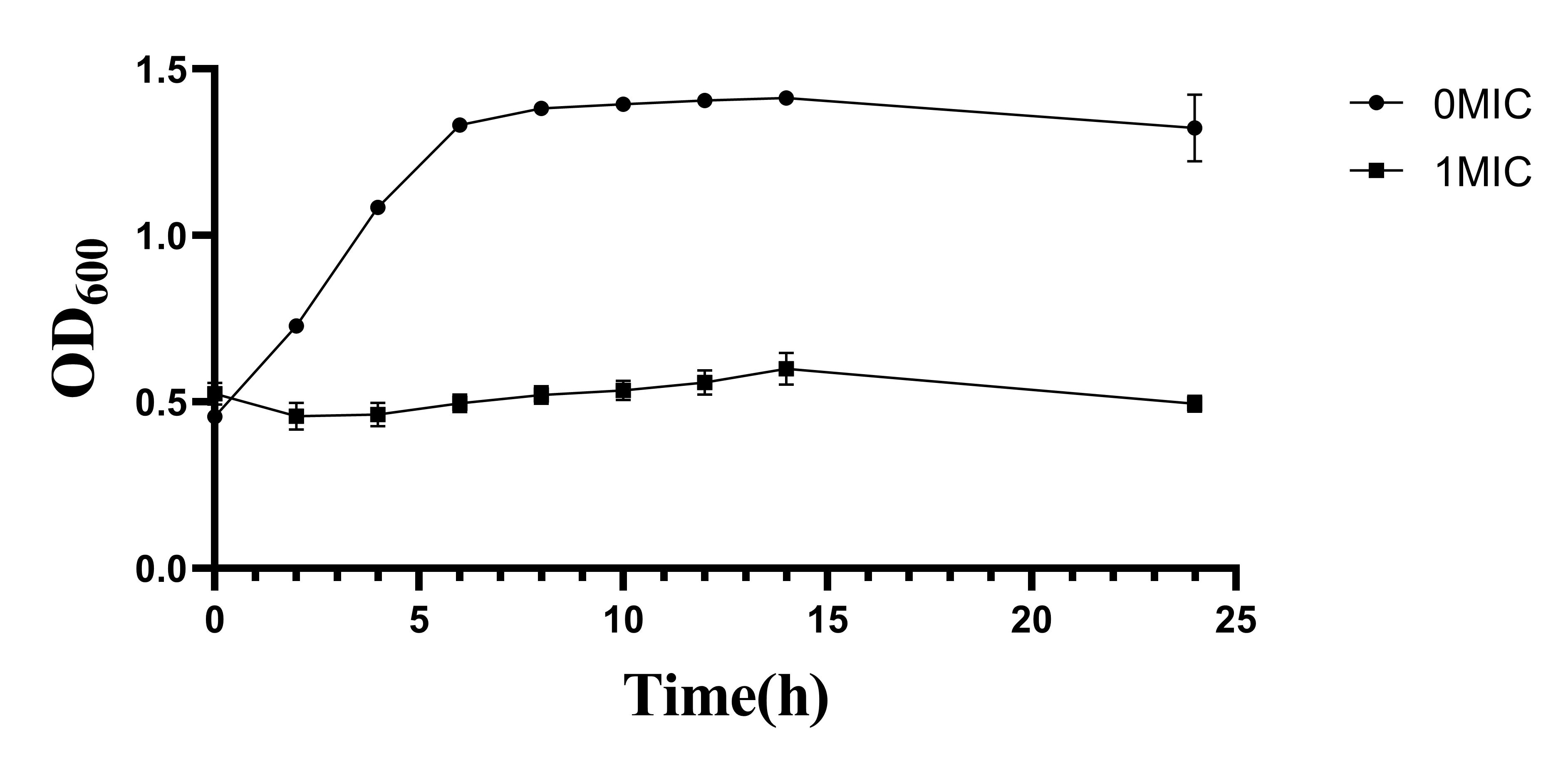

Supplement: Supplementary Figure 1 — Killing-growth curve analysis of C. albicans SC5314 strains in the absence and presence of 1MIC IBC. C. albicans SC5314 (3 replicates) was grown in a deep 24-well plate at 30°C. For determining the growth, 200 μL were aspirated every 2 h and monitored as OD600 for 24 h using the growth reader under constant shaking. The growth curve was prepared based on the growth of three independent cultures, and the mean ± SD was plotted. [file Image_1.jpeg]
